# Supplementary material for: Functional Characterisation of Three Glycine N-Acyltransferase Variants and the Effect on Glycine Conjugation to Benzoyl–CoA
Source: Int J Mol Sci. 2021 Mar 18;22(6):3129. doi: 10.3390/ijms22063129 (PMC8003330; doi:10.3390/ijms22063129)
Supplement: Supplementary file 1 [file ijms-22-03129-s001.zip › Supplementary files/Supplementary Information.pdf]

Supplementary Information

Functional characterisation of three glycine N-acyltransferase variants and the effect on glycine conjugation to benzoyl-CoA

Johann M. Rohwer[1], Chantelle Schutte[2] and Rencia van der Sluis[2]

[1]Laboratory for Molecular Systems Biology, Department of Biochemistry, Stellenbosch University, Private Bag X1, Matieland, Stellenbosch 7602, South Africa

[2]Focus Area for Human Metabolomics, North-West University, Private Bag X6001, Potchefstroom, 2520, South Africa

Supplementary Tables

Supplementary Table 1

Missense variants identified in GLYAT using gnomAD

The table was generated by exporting only the missense variants located in the canonical GLYAT transcript (ENST00000344743.3). The data was sorted according to the allele frequency of all the combined populations.

Filename: Suppl Table 1. gnomAD_v2.1.1_ENST00000344743_2020_09_08_13_34_13_edited.xlsx

Supplementary Table 2

Haplotype frequency data obtained from Ensembl

The table was generated by exporting the haplotype frequencies of the ENST00000344743.8 GLYAT transcript. One haplotype that resulted in a nonsense mutation was excluded from the study.

Filename: Suppl Table 2. Haplotype frequency data obtained from ensemble 20200929

Supplementary Table 3

Initial rate data for the kinetic experiments.

The Excel sheets contain initial rates (absorbance change per minute) for each replicate at the specified glycine and benzoyl-CoA concentrations, for kinetic experiments with the 156Asn>Ser; 17Ser>Thr,156Asn>Ser; and 156Asn>Ser,199Arg>Cys GLYAT haplotype variants. Kinetic assays were performed on microtitre plates as described under Methods in the main paper.

Filename: Suppl Table 3. Initial rate data.xlsx

Supplementary Figures

Supplementary Figures 1 – 6 contain rate-vs.-substrate concentration plots for the kinetic characterizations of the three GLYAT haplotype variants with the global model fit. The data are a replot of Figure 5 in the main paper as two-dimensional scatter plots of rate against one substrate concentration for all the concentrations of the second substrate studied. Note that the line on each plot indicates the global model fit on all the data, evaluated at the relevant substrate concentrations. For individual graphs, discrepancies can thus be explained by the fact that not only the data in that particular plot were taken for the model fit, but all data simultaneously. Also note that while Figure 5 of the main paper plots data points as means of replicates, individual data points are presented here.

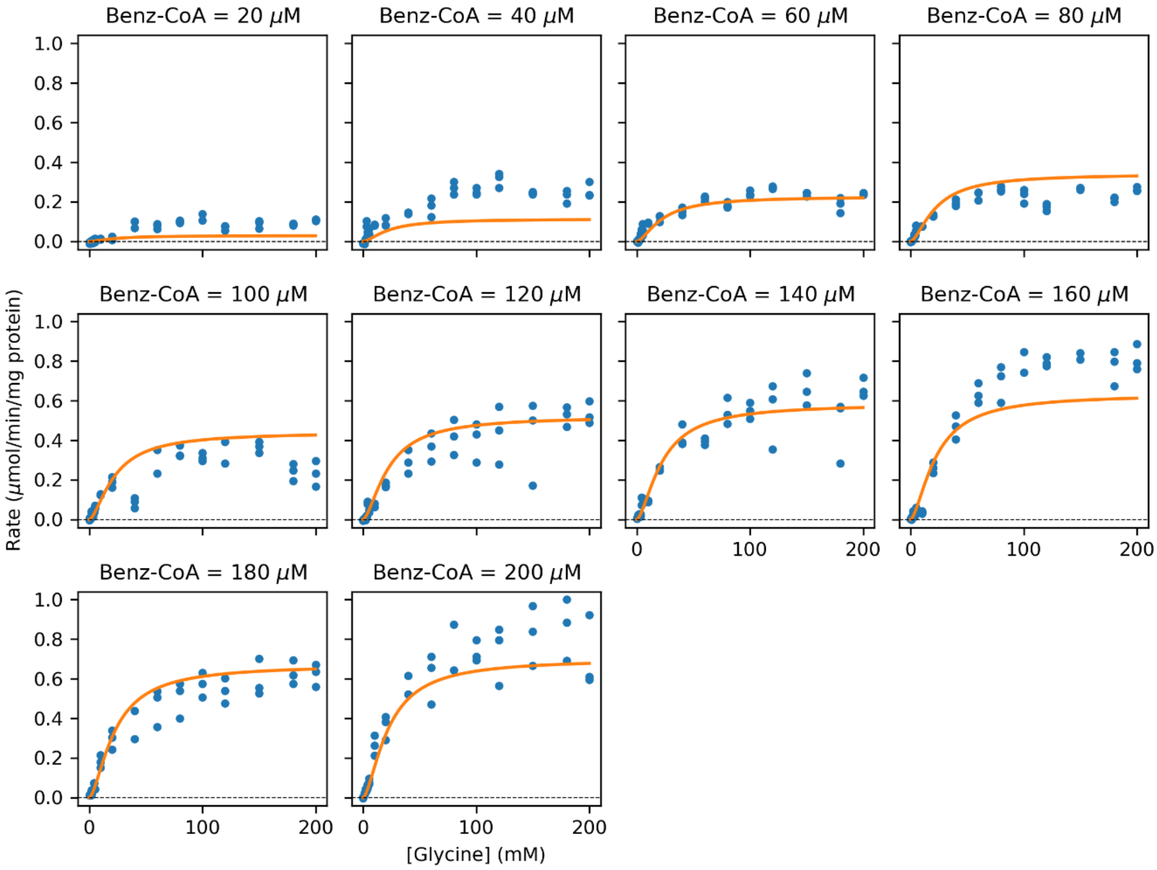

Supplementary Figure 1: Rate-vs.-glycine concentration plots and model fits at varying benzoyl-CoA concentrations for the 156Asn>Ser haplotype variant.

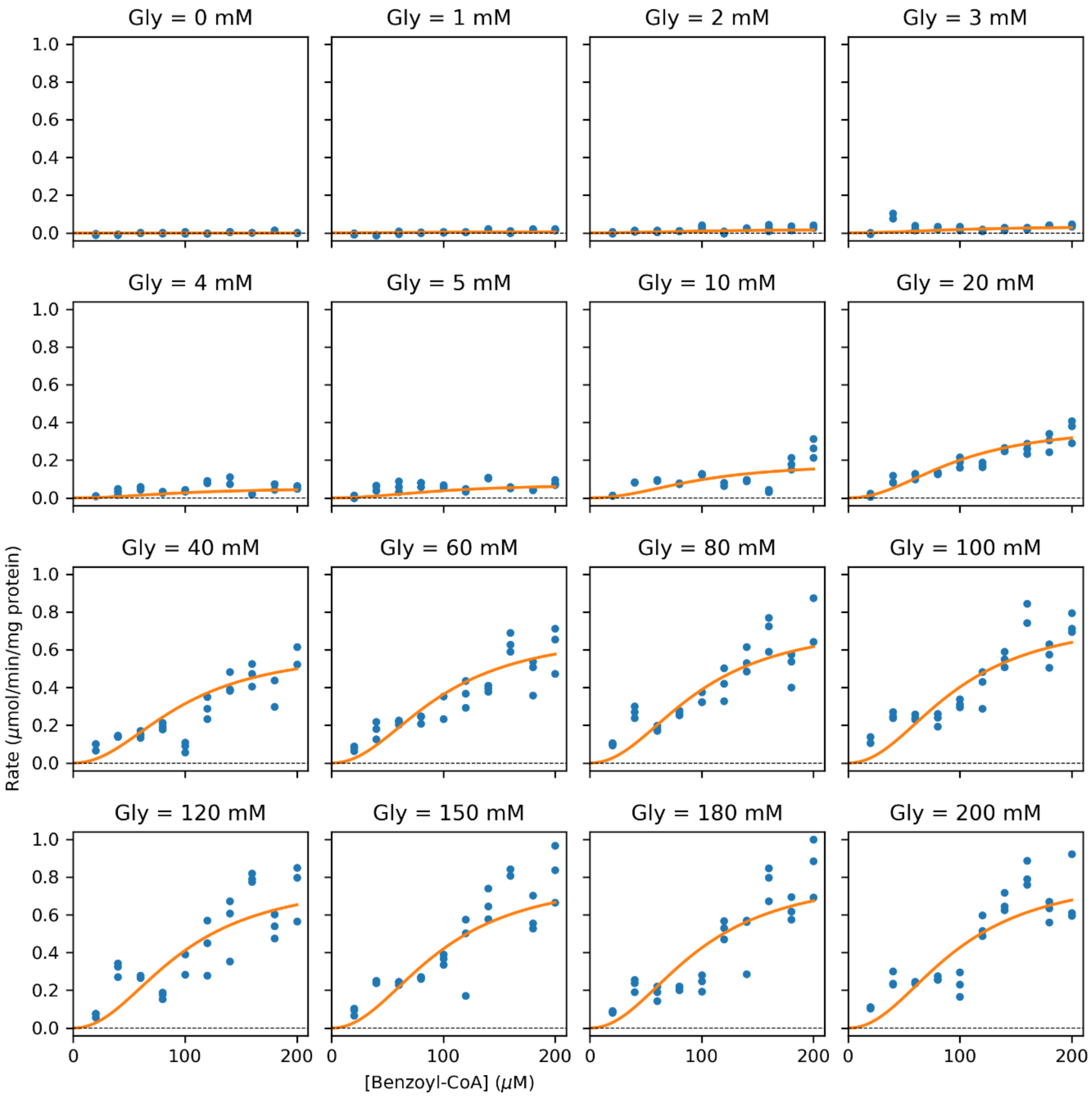

Supplementary Figure 2: Rate-vs.-benzoyl-CoA concentration plots and model fits at varying glycine concentrations for the 156Asn>Ser haplotype variant.

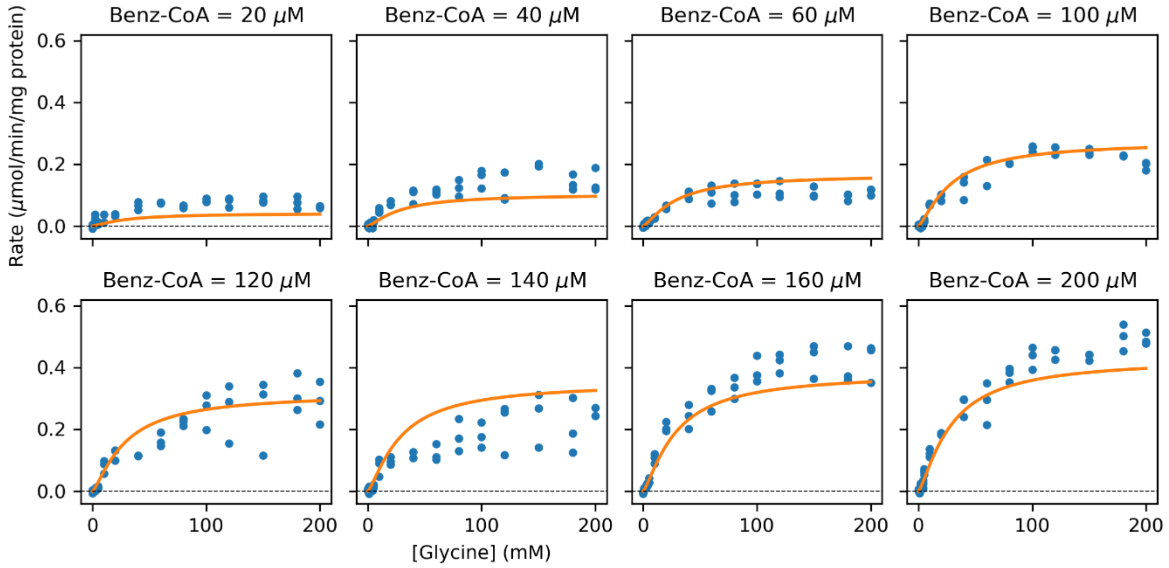

Supplementary Figure 3: Rate-vs.-glycine concentration plots and model fits at varying benzoyl-CoA concentrations for the 17Ser>Thr,156Asn>Ser haplotype variant.

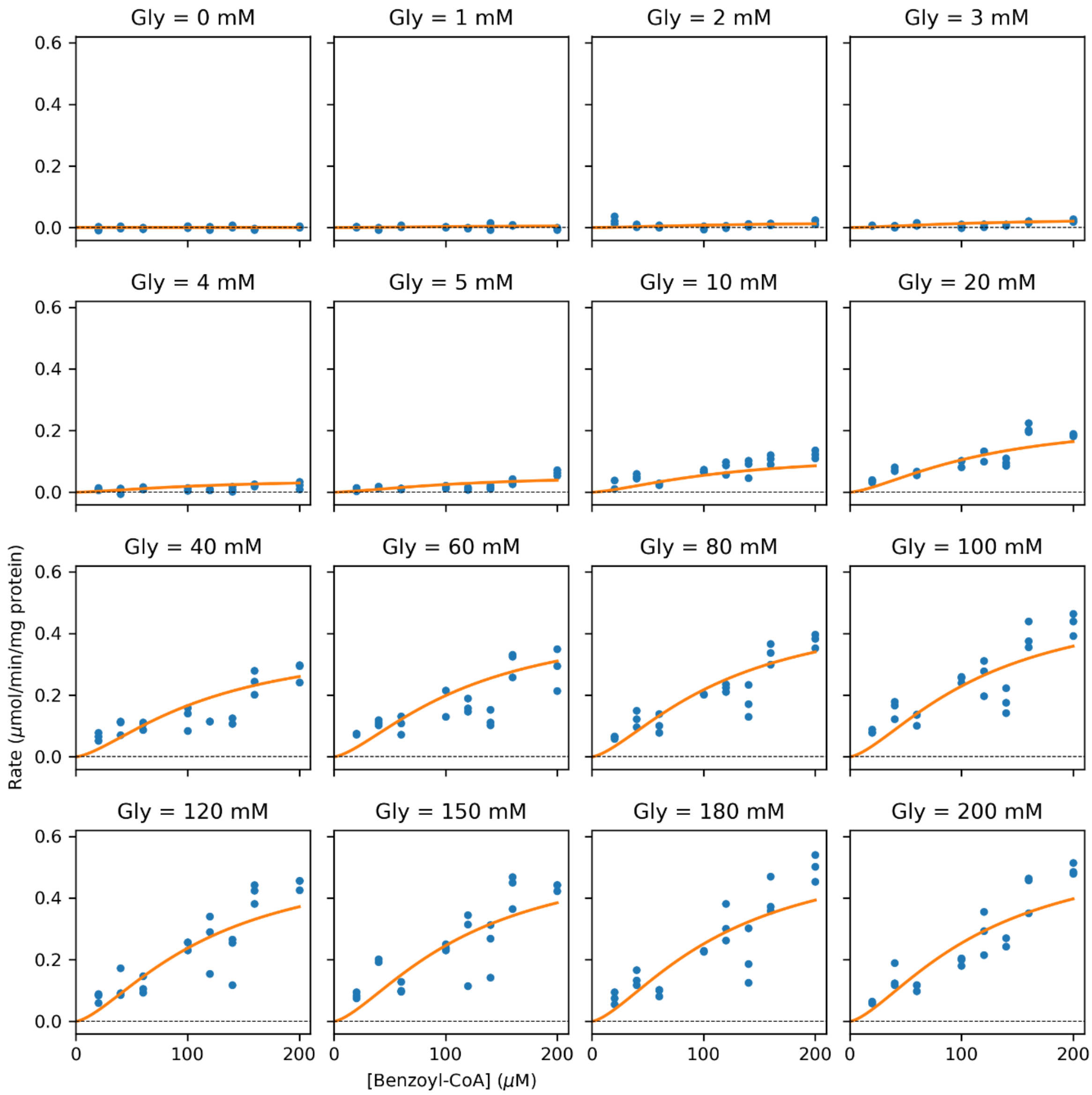

Supplementary Figure 4: Rate-vs.-benzoyl-CoA concentration plots and model fits at varying glycine concentrations for the 17Ser>Thr,156Asn>Ser haplotype variant.

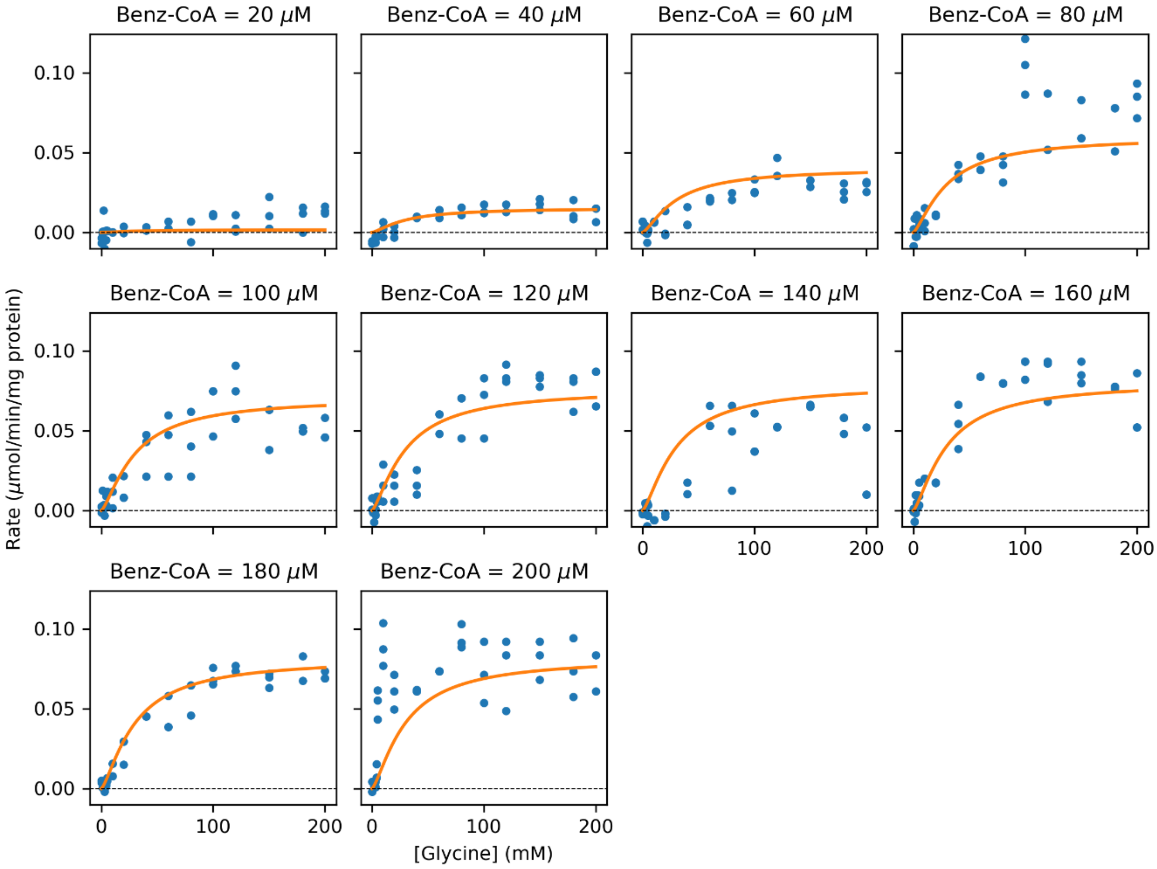

Supplementary Figure 5: Rate-vs.-glycine concentration plots and model fits at varying benzoyl-CoA concentrations for the 156Asn>Ser,199Arg>Cys haplotype variant.

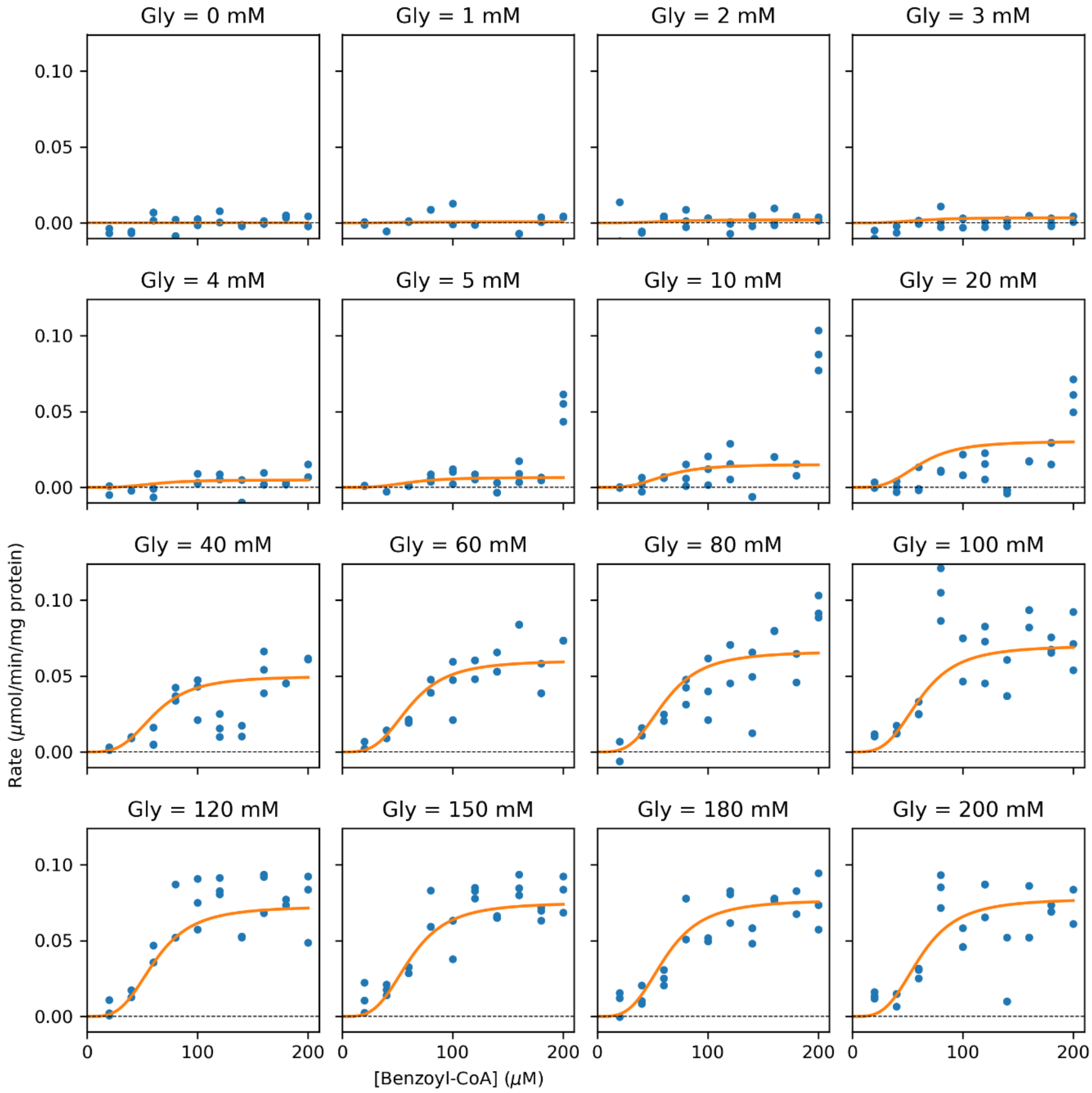

Supplementary Figure 6: Rate-vs.-benzoyl-CoA concentration plots and model fits at varying glycine concentrations for the 156Asn>Ser,199Arg>Cys haplotype variant.

Supplementary Figures 7 – 12 show Lineweaver-Burk plots of selected data (substrate concentrations ≥5 mM) presented in Supplementary Figures 1 – 6 for the kinetic characterizations of the three GLYAT haplotype variants. As previously, the line on each plot indicates the global model fit on all the data, evaluated at the relevant substrate concentrations. Positive cooperativity is indicated by concave curvature of the fitted lines (deviation from linearity). The fit was performed directly on the data, not on the reciprocal data as plotted here. For individual graphs, discrepancies can thus be explained by the fact that not only the data in that particular plot were taken for the model fit, but all data simultaneously. Also note that the Lineweaver-Burk plot distorts and exaggerates any errors at low substrate concentrations (high 1/[S] and 1/*v* values).

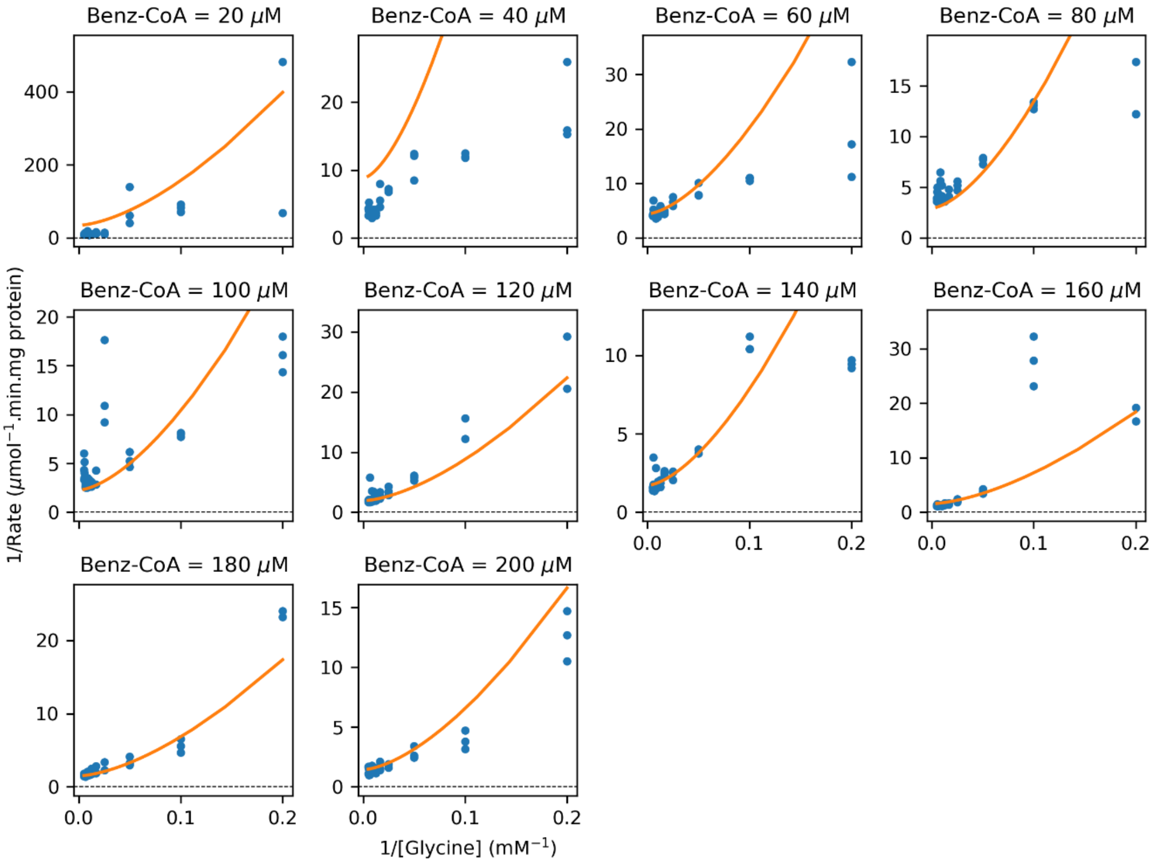

Supplementary Figure 7: Lineweaver-Burk plots for glycine at different benzoyl-CoA concentrations for the 156Asn>Ser haplotype variant.

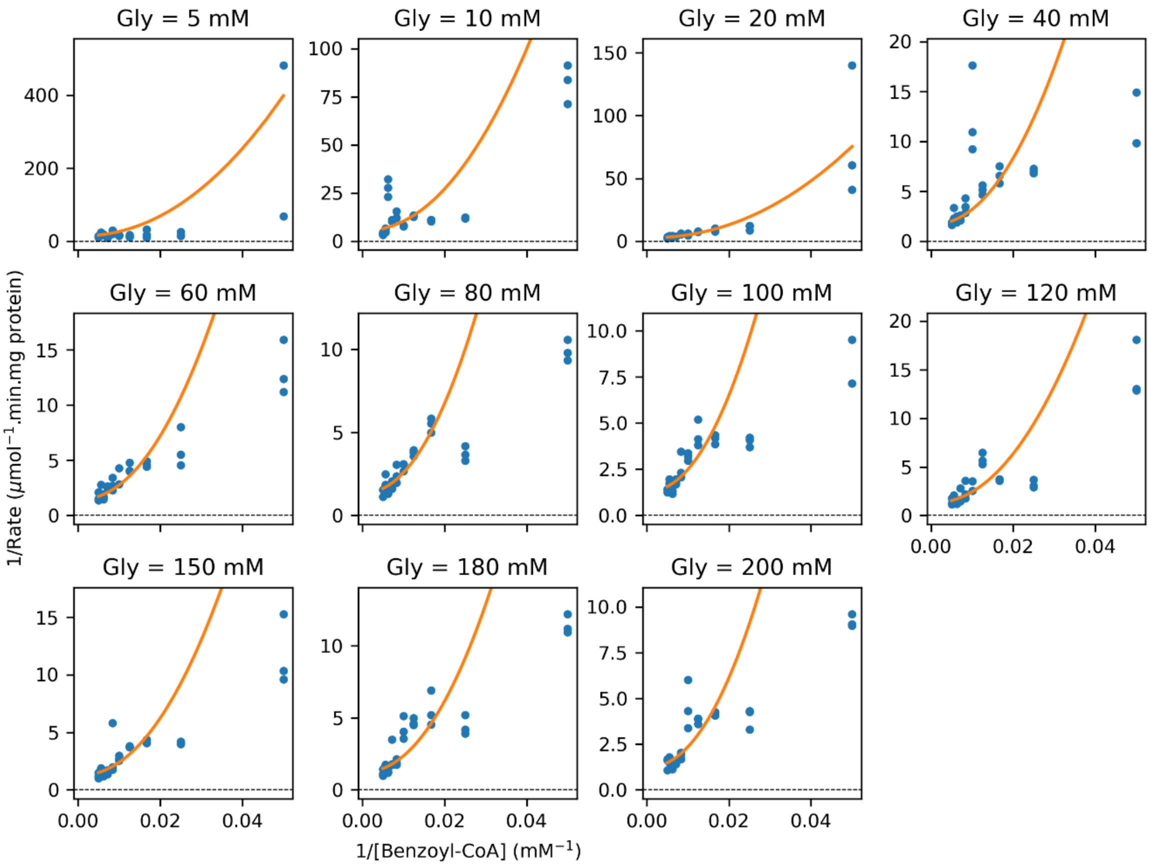

Supplementary Figure 8: Lineweaver-Burk plots for benzoyl-CoA at different glycine concentrations for the 156Asn>Ser haplotype variant.

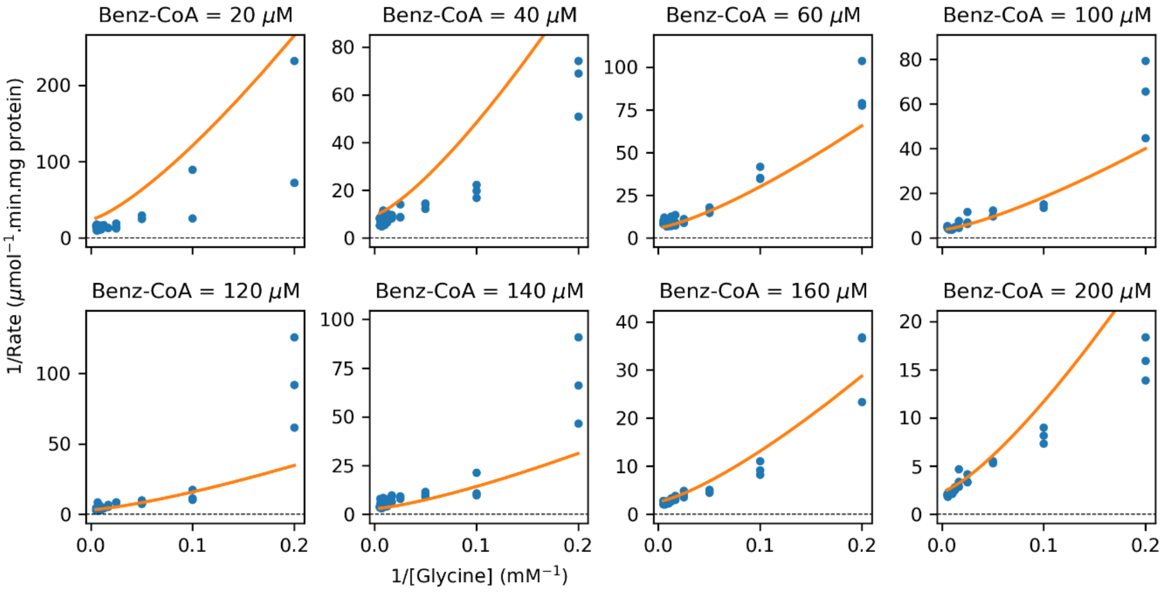

Supplementary Figure 9: Lineweaver-Burk plots for glycine at different benzoyl-CoA concentrations for the 17Ser>Thr,156Asn>Ser haplotype variant.

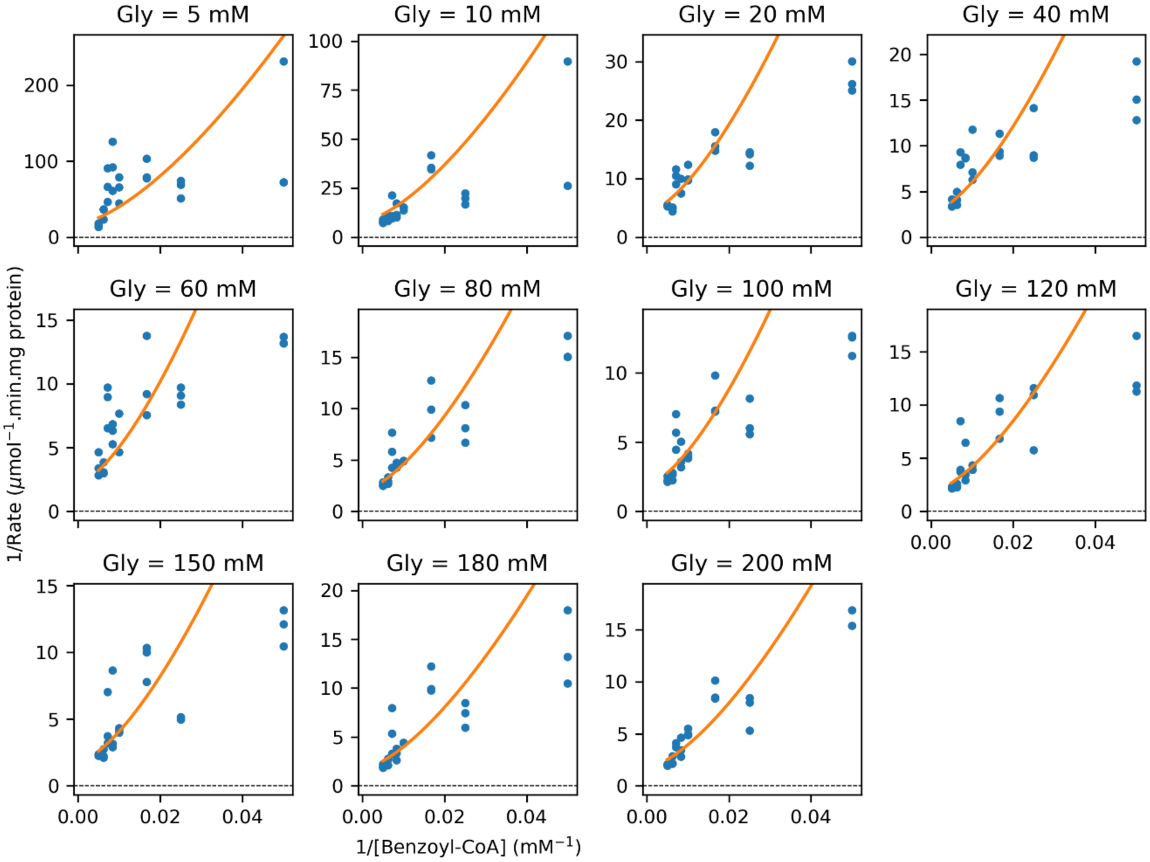

Supplementary Figure 10: Lineweaver-Burk plots for benzoyl-CoA at different glycine concentrations for the 17Ser>Thr,156Asn>Ser haplotype variant.

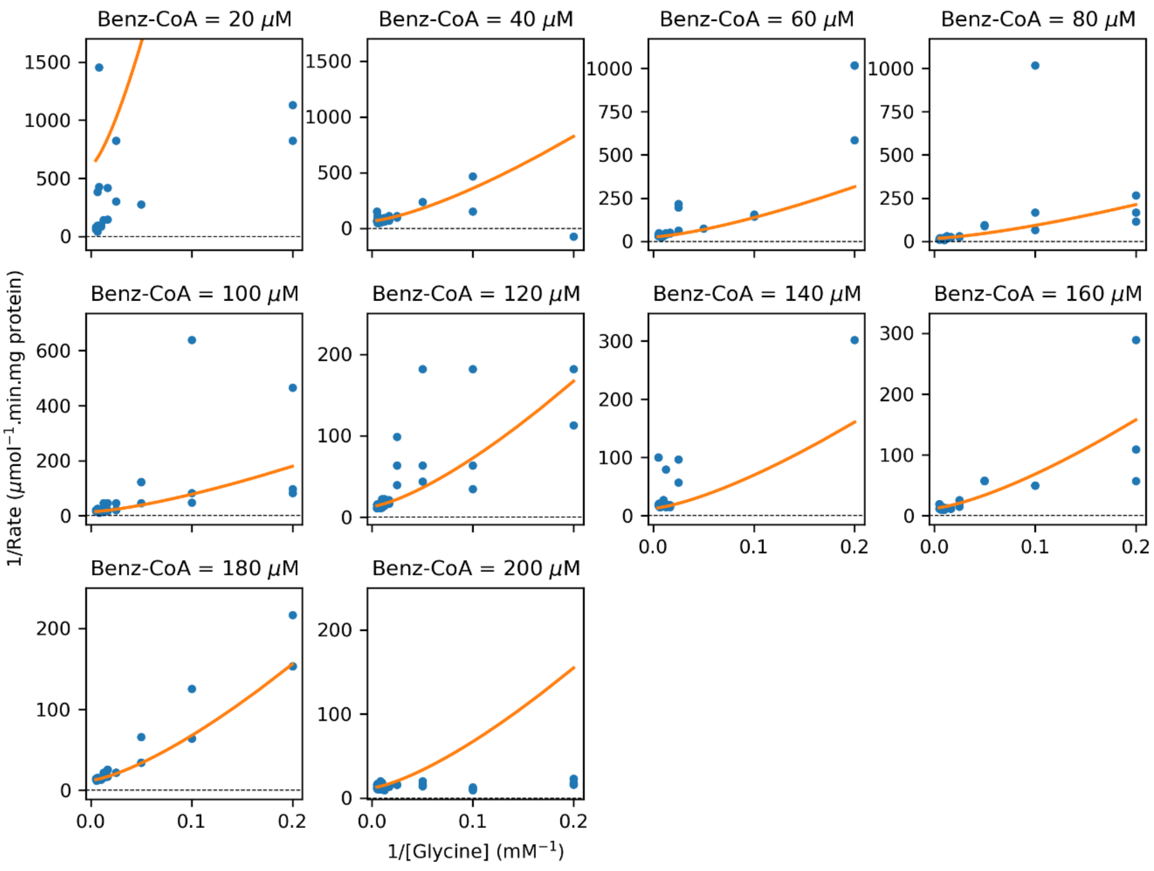

Supplementary Figure 11: Lineweaver-Burk plots for glycine at different benzoyl-CoA concentrations for the 156Asn>Ser,199Arg>Cys haplotype variant.

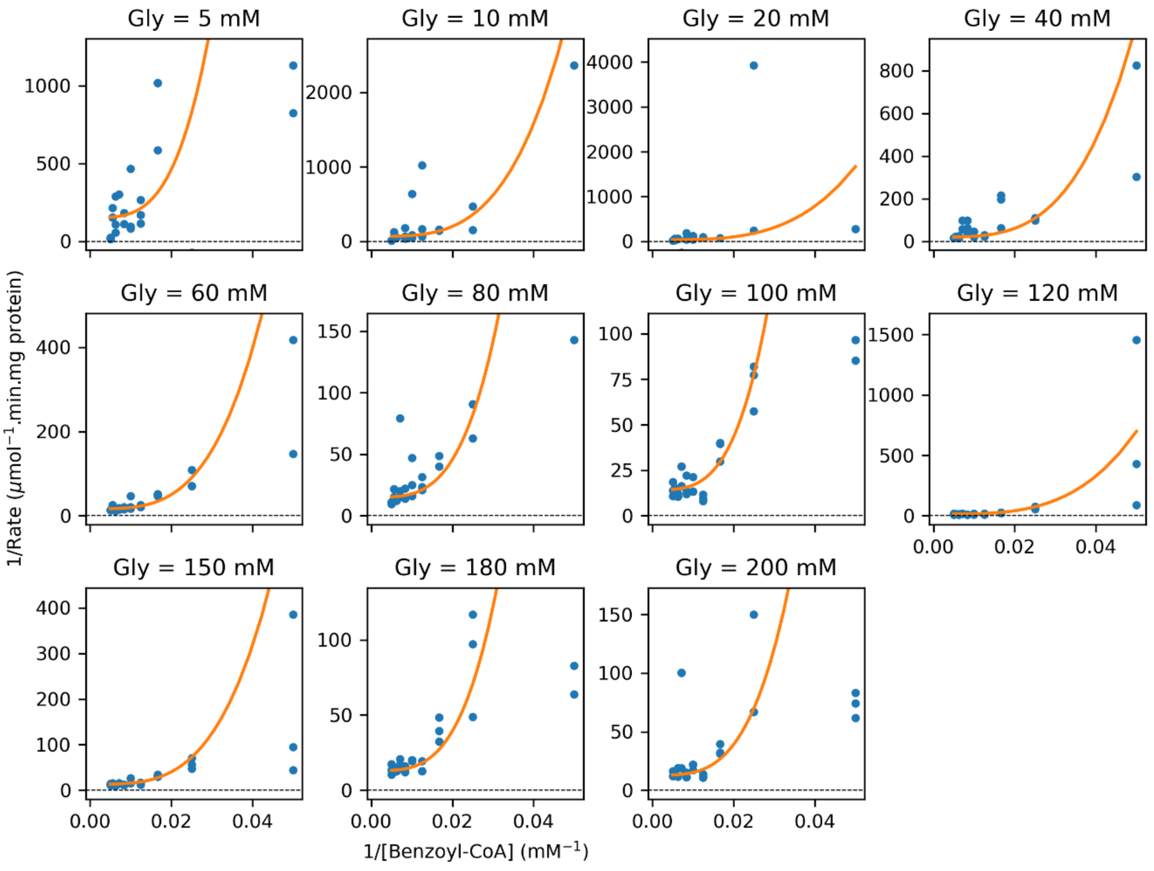

Supplementary Figure 12: Lineweaver-Burk plots for benzoyl-CoA at different glycine concentrations for the 156Asn>Ser,199Arg>Cys haplotype variant.
